# Massive Primary Thyroid Lymphoma on [^18^F]-FDG-PET/CT: A Literature Review of a Rare Case of Rapidly Progressive Goitre

**DOI:** 10.3390/diagnostics15172180

**Published:** 2025-08-28

**Authors:** Ayoub Jaafari, Sébastien Mehaudens, Olivier Gheysens, Sarah Bailly, Nicolas Schobbens, Michel Mourad, François Jamar

**Affiliations:** 1 Nuclear Medicine Department, Cliniques Universitaires Saint-Luc, 1200 Brussels, Belgium; sebastien.mehaudens@saintluc.uclouvain.be (S.M.); olivier.gheysens@saintluc.uclouvain.be (O.G.); francois.jamar@saintluc.uclouvain.be (F.J.); 2 Hematology Department, Cliniques Universitaires Saint-Luc, 1200 Brussels, Belgium; sarah.bailly@saintluc.uclouvain.be (S.B.); nicolas.schobbens@saintluc.uclouvain.be (N.S.); 3 Surgery Department, Cliniques Universitaires Saint-Luc, 1200 Brussels, Belgium; michel.mourad@saintluc.uclouvain.be

**Keywords:** primary thyroid lymphoma, [^18^F]FDG PET/CT, Goitre, rapid tumour progression, hybrid imaging, high-grade B-cell lymphoma, triple-expressor lymphoma

## Abstract

**Background:** Primary thyroid lymphoma (PTL) is an uncommon malignancy that predominantly affects women in their sixth or seventh decade. It is strongly associated with chronic lymphocytic thyroiditis (Hashimoto’s thyroiditis) and other autoimmune conditions. The hallmark clinical feature is a rapidly enlarging thyroid mass, which can quickly cause compressive symptoms such as dysphagia, hoarseness, and dyspnoea. Timely recognition and treatment are essential. [^18^F]fluoro-2-deoxy-D-glucose positron emission tomography/computed tomography ([^18^F]FDG-PET/CT) plays a central role in the diagnosis, staging, response assessment, prognostication, and surveillance of high-grade lymphomas, significantly influencing clinical management. **Case presentation**: We report the case of a woman in her sixties with a history of multinodular goitre but without an autoimmune background, who presented with a large left-sided cervical mass that had rapidly enlarged over approximately two months. Laboratory tests, fine-needle aspiration (FNA), and [^18^F]FDG-PET/CT revealed abnormal cytology and a highly hypermetabolic necrotic left thyroid mass, without extra-thyroidal disease, suggestive of lymphoma. Definitive biopsy with immunohistochemistry confirmed a high-grade B-cell lymphoma, positive for CD5 and demonstrating triple expression of Bcl2, Bcl6, and c-Myc. The patient underwent chemotherapy, achieving a marked morphometabolic response after two cycles, consolidated after four cycles. **Conclusions**: This rare case highlights the importance of considering PTL in the differential diagnosis of an isolated, rapidly enlarging thyroid mass, regardless of prior Hashimoto’s thyroiditis. Early diagnosis and timely treatment are crucial to improve patient outcomes.

## 1. Introduction

Primary thyroid lymphoma (PTL) is a rare malignancy, accounting for approximately 2–5% of all thyroid cancers and only 2% of extra-nodal lymphomas [1]. Among PTLs, diffuse large B-cell lymphoma (DLBCL) is the most common subtype, representing 50–80% of the cases [2]. This entity predominantly affects elderly individuals, particularly women, and is strongly associated with chronic lymphocytic thyroiditis (Hashimoto’s thyroiditis) [3]. Notwithstanding its rarity, thyroid DLBCL is clinically relevant due to its rapid progression, compressive symptoms, and potential for airway compromise, necessitating prompt recognition and treatment. Clinically, patients often present with a rapidly (several weeks) enlarging thyroid mass, sometimes accompanied by dysphagia, hoarseness, dyspnoea, and B symptoms (fever, night sweats, and weight loss). It evolves more quickly than the most frequent papillary and follicular thyroid carcinomas, and conversely less abruptly than most of the invasive anaplastic thyroid carcinomas (ATC) [4]. In contrast to the latter, thyroid DLBCL is highly responsive to chemotherapy and has a relatively better prognosis when diagnosed early [2]. Imaging techniques such as ultrasound, CT, and [^18^F]FDG-PET/CT play a crucial role in identifying PTL, but definitive diagnosis requires histopathological confirmation through core-needle biopsy or surgical biopsy with immunohistochemistry [5]. Herein, we describe a rare case of a patient with primary thyroid DLBCL with a history of toxic multinodular goitre but without Hashimoto’s thyroiditis, highlighting the diagnostic challenges, clinical presentation, and therapeutic considerations based on a comprehensive review of the literature.

## 2. Case Presentation

**Figure 1:**
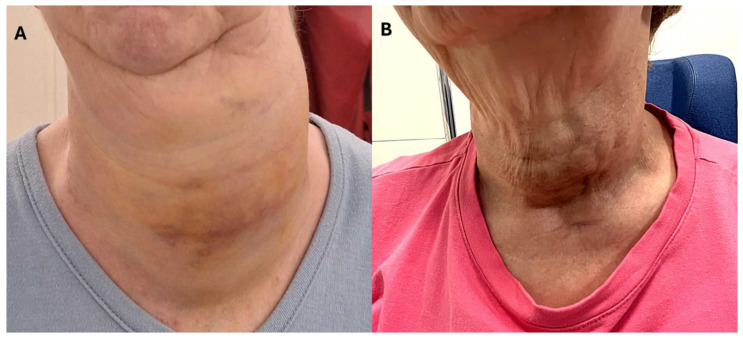
(**A**) A clinical photograph of the patient’s neck showing a firm, left-sided cervical mass measuring approximately 12 cm in its greatest dimension, with overlying skin retraction, ecchymotic discolouration, and prominent dilated superficial veins and telangiectasias. (**B**) A follow-up image (end-of-treatment) demonstrating marked regression of the indurated mass to around 3 cm, with only subtle skin retraction remaining.

A 63-year-old female patient was referred to our institution at the end of November 2024 by her general practitioner for a large left cervical mass that had been growing in size for a relatively short time (Figure 1A). The patient had been diagnosed with multinodular goitre in 2012 and reported a marked increase in the size of her goitre since October 2024, with continuous progression up to the current consultation. The patient also described increasing difficulty eating, caused by the mass, which has led to a weight loss of 1 to 1.5 kg in recent weeks. Despite these symptoms, the patient reported no breathing complaints, no hoarseness, no fever, and/or no recent infection. There were no obvious B signs. The patient’s medical history included a well-known toxic multinodular goitre since 2012 and hyperthyroidism, initially treated with propylthiouracil 50 mg daily, chronic post-traumatic back pain, asthma, and hypertension, all under control with appropriate treatments. No relevant family history was reported. On clinical examination of the neck, a large left-sided cervical hard mass was palpable, measuring approximately 12 cm, craniocaudal, with skin retractions and superficial vein dilation/telangiectasias, and no cervical or extra-cervical lymphadenopathy. The weight was 53.8 kg, with a blood pressure of 120/80 mmHg, heart rate of 99 bpm, and oxygen saturation of 100% on room air. Her general condition was considered normal, with no signs of excessive fatigue and an overall normal appearance. Cardiovascular, pulmonary, abdominal, and neurological examination were unremarkable, and no peripheral oedema was noted.

Bearing in mind the patient’s condition at the time of consultation, further investigations were carried out. A blood test showed haemoglobin at 11.9 g/dL (N: 12.2–15.0 g/dL), normocytic normochromic, platelets at 187.000/µL (N: 150–450 × 10^3^/µL), white blood cells at 4.830 × 10^3^/µL (N: 4.000–6.000 × 10^3^/µL) with absolute neutrophils at 3.390 × 10^3^/µL (N: 1.600–7.00 × 10^3^/µL) and lymphocytes at 0.83 × 10^3^/µL (N: 0.800–5.00 × 10^3^/µL), and normal coagulation, ionogram, renal, and liver functions. Thyroid function tests showed a low TSH at 0.09 mU/L (N: 0.27–4.60 mU/L), free T4 decreased to 11.0 (N: 12.0–22.0 pmol/L), and a thyroglobulin increased to 218.6 (N < 40.0 ng/mL), without TPO and TSH-R antibodies (Anti-TSH-R). Lymphocyte typing revealed T lymphopenia with normal CD4 and CD8 T lymphocyte counts, with no impact on the CD4/CD8 ratio, and normal NK lymphocyte levels. A fine needle aspiration biopsy (FNAB) of the thyroid mass revealed abnormal cytology suggestive of lymphoma, although the exact histological type could not be determined from this sample. The lesion was classified as category 6 according to the Bethesda system (2008), indicating an almost certain diagnosis of malignancy.

**Figure 2:**
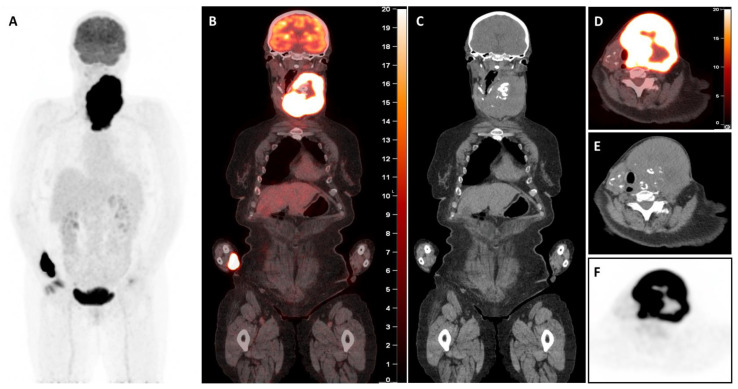
Baseline [^18^F]-fluorodeoxyglucose positron emission tomography/computed tomography ([^18^F]FDG-PET/CT) images. The whole-body maximum intensity projection (MIP, **A**) image shows a large left mass area of abnormal tracer uptake in the neck and normal tracer distribution in the rest of the body. Coronal (**B**,**C**) and axial (**D**–**F**) merged PET/CT section shows gross enlargement of the thyroid gland with a necrotic centre, calcifications, and displacing trachea to the right with no evidence of compression or infiltration. The mass predominantly involves the left thyroid lobe along with part of the isthmus. Intense [^18^F]-FDG uptake (SUVmax 19.4) in the peripheral part of the thyroidal mass is seen with no tracer uptake in the necrotic centre (**B**,**D**,**F**).

**Figure 3:**
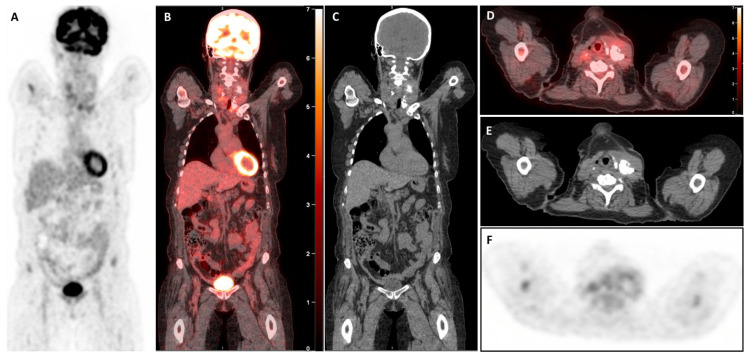
Interim [^18^F]FDG-PET/CT (iPET) images after two cycles of chemotherapy. The whole-body MIP (**A**) image shows a normal distribution of tracer throughout the body, associated with diffuse axial and appendicular skeletal activity of reactive nature, and a substantial absence of significant tracer activity in the neck. Coronal (**B**,**C**) and axial (**D**–**F**) unenhanced CT images of the neck reveal significant reduction in the size of the thyroid gland with calcifications in the left thyroid lobe (craniocaudal dimension from 14 cm to 10 cm). Subsequent [^18^F]FDG-PET/CT images show diffuse [^18^F]-FDG uptake in the residual thyroidal mass (SUVmax 3.0) less than hepatic uptake (SUVmax 3.1), suggestive of a complete metabolic response (Deauville score of 3).

**Figure 4:**
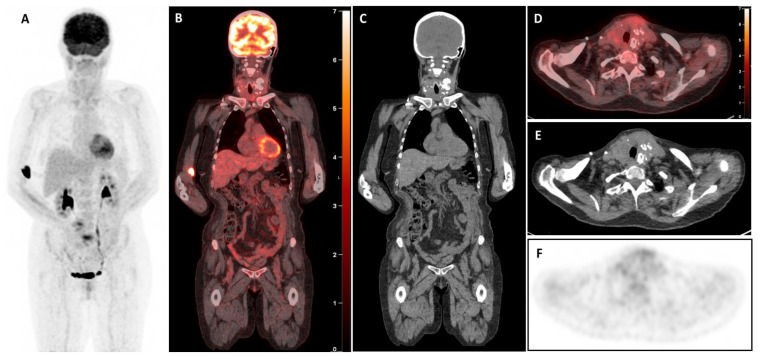
Interim [^18^F]FDG-PET/CT images after four cycles of chemotherapy demonstrated normal tracer distribution on the whole-body MIP image (**A**), with diffuse axial and appendicular skeletal uptake consistent with reactive post-chemotherapy changes, and no abnormal tracer activity was noted in the neck region on the coronal projection (**B**,**C**). Notably, the [^18^F]-FDG PET/CT revealed no pathological uptake in the residual thyroid mass (SUVmax 2.7) (**D**–**F**), lesser than the liver blood pool (SUVmax 3.1) consistent with a complete metabolic response (Deauville score 3).

**Figure 5:**
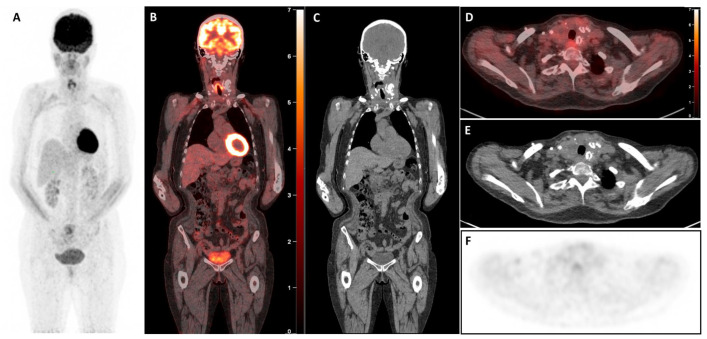
End-of-treatment [^18^F]FDG PET/CT showed normal tracer distribution on the whole-body MIP image (**A**), with no significant pathological uptake in the residual thyroid mass. The lesion demonstrated an SUVmax of 2.9, which was lower than hepatic uptake (SUVmax 3.3) but higher than the mediastinal blood pool (SUVmax 1.9), consistent with a complete metabolic response (Deauville score 3). (**B**,**C**) and (**D**–**F**) are coronal and axial images, respectively. B and D are PET and CT fused images.

The [^18^F]FDG-PET/CT revealed a large, highly hypermetabolic thyroid mass, 140 mm in craniocaudal length, with a necrotic component and multiple macro-calcifications, extending from the skin to the prevertebral tissues (antero-posterior axis) and from the submandibular region to the anterior mediastinum, reaching the mid-manubrium (craniocaudal axis). The mass caused rightward deviation and compression of the trachea, reducing its transverse diameter to only 7 mm, with no evidence of extra-thyroidal involvement (Figure 2). A biopsy of this mass was performed within a few days, and pathology and immunohistochemical examination confirmed the presence of a high-grade B-cell lymphoma, expressing CD5+ and showing triple expression of Bcl2, Bcl6, and c-Myc. The lymphoma was classified as non-germinal centre (non-GC), with no rearrangement detected in FISH tests and stage IE (bulky). The patient started chemotherapy with polatuzumab-R (rituximab) and CHP (doxorubicin, cyclophosphamide, methylprednisolone), which was well tolerated. Furthermore, considering the potential risk of agranulocytosis, the treatment for hyperthyroidism was modified by transitioning from propylthiouracil to methimazole at a dosage of 10 mg per day. Interim [^18^F]FDG-PET/CT (iPET) performed after two cycles of chemotherapy (Figure 3) demonstrated a complete metabolic response (Deauville score 3), accompanied by substantial morphological regression of the large thyroid mass. The [^18^F]FDG-PET/CT performed after four cycles (Figure 4) and at the end of treatment (Figure 5) confirmed the persistence of a complete metabolic response, while a calcified residual lesion, measuring 2.5 cm in craniocaudal extent, remained visible, correlating well with the clinical appearance of the neck (Figure 1B).

## 3. Discussion

Primary thyroid lymphoma (PTL) is a rare malignancy, typically affecting females in their sixth and seventh decades of life, and is strongly associated with Hashimoto’s thyroiditis (HT) in approximately 80% of cases [1,6]. In contrast, multinodular goitre (MNG) is a common benign condition marked by the presence of multiple nodules within an enlarged thyroid gland. A comprehensive review of the extant medical literature reveals limited evidence that directly associates PTL with MNG [7]. While both conditions involve thyroid enlargement and nodularity, their underlying pathophysiological mechanisms differ significantly. PTL has been linked to autoimmune processes, while MNG has been associated with non-autoimmune factors, such as iodine deficiency [8,9]. However, the literature underscores that any thyroid gland with a multinodular appearance that suddenly shows signs of rapid growth or compressive symptoms should be carefully evaluated for the possibility of PTL—even in the setting of multinodular goitre as in our case [10].

The pathogenesis of PTL is not entirely understood. In general, the thyroid gland does not contain lymphoid tissue. However, under pathological conditions, the presence of lymphocytes may be observed, thereby promoting the further development of the disease [11]. Chronic antigenic stimulation from autoimmune thyroiditis is considered a significant risk factor. Patients with HT have a markedly increased risk of developing PTL, estimated to be 40 to 80 times greater than that of the general population [12]. Although HT is a major risk factor, PTL can occur in patients without a history of autoimmune thyroiditis, suggesting alternative pathogenic mechanisms. Chronic antigenic stimulation in the absence of HT could be driven by other sources of chronic immune stimulation, such as infections, environmental factors, mutation in genes regulating the NF-κB pathway, and genetic factors which might contribute to lymphomagenesis [13,14]. However, specific associations have yet to be clearly identified.

The clinical presentation of PTL typically involves a rapidly enlarging thyroid mass, leading to compressive symptoms such as dysphagia, hoarseness, and dyspnoea. These manifestations necessitate prompt medical evaluation due to the potential for airway compromise [15]. In our case, the patient experienced significant cervical swelling and dysphagia, consistent with these typical presentations.

Differential diagnosis may include intra-nodular haemorrhage, an acute or subacute infectious process (e.g., thyroid tuberculosis), unilateral subacute (de Quervain’s) thyroiditis, primary sarcoma, and the dreadful anaplastic thyroid carcinoma. Classical differentiated thyroid carcinoma is unlikely to evolve as rapidly, and Riedel’s thyroiditis can be impressive but usually progresses slowly [3]. In the presented case, metastasis from another solid tumour was deemed unlikely in the absence of personal history of previous cancer.

Imaging plays a pivotal role in the diagnosis, staging, and management of PTL, providing crucial insights into tumour characterisation and treatment response [16]. Among available modalities, [^18^F]FDG-PET/CT is particularly valuable in differentiating PTL from secondary thyroid involvement by systemic lymphoma. PTL typically appears as a solitary, intensely FDG-avid lesion within the thyroid, whereas widely spread lymphoma demonstrates more heterogeneous and less intense uptake in the thyroid, reflecting secondary infiltration [17]. PET-CT not only aids in the diagnosis but also assesses disease extent, detects systemic involvement, and monitors treatment response. In PTL, metabolic activity is largely confined to the thyroid with minimal regional lymph node spread, while metastatic lymphoma frequently exhibits diffuse FDG-avid lesions across multiple organ systems [18]. Additionally, PET-CT plays a key role in biopsy guidance, therapy evaluation using the Deauville scoring system, and long-term disease surveillance, making it an indispensable tool in clinical decision-making [19].

While FNA can suggest lymphoma, definitive diagnosis requires core-needle or surgical biopsy with immunohistochemical analysis [20]. In our patient, FNA indicated abnormal cytology, and subsequent biopsy confirmed a high-grade B-cell lymphoma expressing CD5 and triple expression of Bcl2, Bcl6, and c-Myc.

High-grade B-cell lymphomas expressing Myc, Bcl2, and Bcl6 proteins, known as “triple expressors,” are associated with aggressive behaviour and poor prognosis. Studies have shown that these lymphomas exhibit inferior survival rates when treated with standard R-CHOP chemotherapy [21,22]. In this case, the patient received an intensified treatment regimen comprising polatuzumab-R (rituximab) combined with CHP (cyclophosphamide, doxorubicin, methylprednisolone). This approach led to a spectacular complete metabolic response (Deauville score 3), accompanied by substantial morphological regression of the large thyroid mass (iPET, Figure 2). At the end of treatment, the [^18^F]FDG-PET/CT confirmed the sustained complete metabolic response, although a calcified residual lesion measuring 2.5 cm in craniocaudal extent remained visible (Figure 1B, Figure 4 and Figure 5). This outcome aligns with emerging data suggesting that intensified regimens may improve the results in high-risk lymphomas.

The patient’s clinical condition has markedly improved, and her follow-up to date has shown an excellent therapeutic response with ongoing remission. Given the patient’s complete metabolic response to polatuzumab-R-CHP and the absence of extra-thyroidal disease, the prognosis is regarded as being cautiously favourable. Nevertheless, this histologic subtype remains associated with a higher risk of relapse and inferior survival relative to standard DLBCL. Consequently, close surveillance is warranted.

## 4. Conclusions

Primary thyroid lymphoma (PTL) is a rare, aggressive malignancy, primarily affecting elderly women and often associated with Hashimoto’s thyroiditis. This case highlights the importance of considering PTL in patients with rapidly enlarging thyroid masses, even in the absence of Hashimoto’s thyroiditis. Imaging with [^18^F]FDG-PET/CT serves as an invaluable tool, offering high sensitivity to detect metabolic activity, assess the extent of the disease, guide biopsies, and monitor treatment responses while also identifying recurrences. Given the aggressive nature of PTL, early and accurate recognition becomes crucial, with a multidisciplinary approach combining PET/CT findings and histopathological and immunohistochemical analyses being essential for precise diagnosis and the development of a tailored treatment plan that can significantly improve prognosis in this rare condition.

## Data Availability

The data used and analysed in this study are available from the corresponding author on reasonable request.
